# Effect of NFX-179 MEK inhibitor on cutaneous neurofibromas in persons with neurofibromatosis type 1

**DOI:** 10.1126/sciadv.adk4946

**Published:** 2024-05-01

**Authors:** Kavita Y. Sarin, Mark Bradshaw, Chris O'Mara, Jahanbanoo Shahryari, John Kincaid, Steven Kempers, John H. Tu, Sunil Dhawan, Janet DuBois, David Wilson, Patrice Horwath, Mark P. de Souza, Christopher Powala, Gerd G. Kochendoerfer, Scott R. Plotkin, Guy F. Webster, Lu Q. Le

**Affiliations:** ^1^ Department of Dermatology, Stanford University Medical Center, Stanford, CA, USA.; ^2^ NFlection Therapeutics, Boston, MA, USA.; ^3^ Minnesota Clinical Study Center, New Brighton, MN, USA.; ^4^ Skin Search of Rochester, Inc., Rochester, NY, USA.; ^5^ Center for Dermatology Clinical Research Inc., Fremont, CA, USA.; ^6^ DermResearch Inc., Austin, TX, USA.; ^7^ The Education and Research Foundation Inc., Lynchburg, VA, USA.; ^8^ Department of Dermatology, UT Southwestern Medical Center, Dallas, TX, USA.; ^9^ Department of Dermatology, University of Virginia School of Medicine, Charlottesville, VA, USA.

## Abstract

This phase 2a trial investigated the efficacy of NFX-179 Topical Gel, a metabolically labile MEK inhibitor, in the treatment of cutaneous neurofibromas (cNFs) in neurofibromatosis type 1. Forty-eight participants were randomized to four treatment arms: NFX-179 Topical Gel 0.05%, 0.15%, and 0.5% or vehicle applied once daily to five target cNFs for 28 days. Treatment with NFX-179 Topical Gel resulted in a dose-dependent reduction in p-ERK levels in cNFs at day 28, with a 47% decrease in the 0.5% NFX-179 group compared to the vehicle (*P* = 0.0001). No local or systemic toxicities were observed during the treatment period, and systemic concentrations of NFX-179 remained below 1 ng/ml. In addition, 20% of cNFs treated with 0.5% NFX-179 Topical Gel showed a ≥50% reduction in volume compared to 6% in the vehicle group by ruler measurement with calculated volume (*P* = 0.021). Thus, NFX-179 Topical Gel demonstrated significant inhibition of MEK in cNF with excellent safety and potential therapeutic benefit.

## Teaser

A phase 2a study of NFX-179 demonstrates dose-dependent p-ERK suppression and reduction in cutaneous neurofibroma volume in NF1.

## INTRODUCTION

Neurofibromatosis type 1 (NF1) is a rare tumor-predisposition syndrome that affects approximately 1 in 3500 persons and is caused by heterozygous loss of the *NF1* gene (*1*). Persons with NF1 are at increased risk of developing skin, eye, and nervous system tumors as well as other complications (*2*). One of the most common manifestations of NF1 is the development of benign skin tumors called cutaneous neurofibromas (cNFs), which are composed of neoplastic Schwann cells, fibroblasts, and mast cells. cNFs occur in virtually all persons with NF1, can number in the thousands, and are a major cause of morbidity, including disfigurement, anxiety, itch, and pain (*3*–*5*). Currently, there are no U.S. Food and Drug Administration (FDA)–approved medical therapies to treat cNFs, and symptomatic lesions are primarily removed through surgical procedures such as excision, electrodesiccation, or ablative laser therapy (*6*–*8*). However, these procedures are painful, time-consuming, result in scarring, and do not prevent tumor recurrence. Thus, there is an imminent need for medical therapies for the treatment of cNFs.

A recent study assessed the perspective of 548 adults with NF1 (*4*). Sixty percent of responders reported feeling very much or extremely bothered by the size, number, location, and appearance of their cNFs. More than 75% of responders stated that a 33 to 66% reduction in cNF volume would have a clinically meaningful impact on their life. The majority of participants felt that a partial response or no further growth would be an acceptable outcome of treatment.

The RAS/mitogen-activated protein kinase (MAPK) pathway plays a critical role in cellular proliferation, differentiation, and survival and has emerged as a promising therapeutic target for cNFs. The neurofibromin protein functions as a tumor suppressor by negatively regulating RAS activity and preventing the downstream activation of the RAS/MAPK pathway (*9*, *10*). Biallelic loss of neurofibromin leads to uninhibited activation of RAS/MAPK signaling, resulting in the development of cNF tumors.

MAPK kinase (MEK) is a kinase downstream of RAS in the MAPK pathway that activates extracellular signal–related kinase (ERK) through phosphorylation. Preclinical studies have shown that orally administered MEK inhibitors effectively suppress MAPK signaling and reduce the size of plexiform and sub-cNFs in NF1 mouse models (*11*–*13*). Clinical trials have also demonstrated the efficacy of MEK inhibitors, such as selumetinib, in reducing tumor volume and improving symptoms in individuals with plexiform neurofibromas (*14*, *15*). Although orally administered MEK inhibitors have shown promise in treating NF1-related tumors, their systemic toxicities and the nonspecific distribution to other organs raise concerns about their long-term safety for the chronic treatment of cNFs (*16*, *17*).

To address these concerns, we developed a metabolically labile MEK inhibitor, NFX-179, that can be delivered topically to cNFs, providing localized and sustained inhibition of MAPK signaling in the target cNF tumor, but is rapidly metabolized in circulation to minimize systemic exposure and adverse effects. We show that NFX-179 Topical Gel can penetrate human skin and inhibit MEK and the downstream MAPK pathway in cNFs ex vivo. We then present the results of the first-in-human phase 2a trial of NFX-179 Topical Gel demonstrating safety, suppression of the p-ERK biomarker in vivo, minimal systemic exposure, and early clinical efficacy in reducing cNF volume.

## RESULTS

### Discovery of NFX-179 as a metabolically labile (“soft drug”) MEK inhibitor

We conducted a drug discovery program to synthesize MEK inhibitors that would be optimal for topical delivery as previously described (*18*). More than 100 compounds were synthesized and screened for potency and selectivity for MEK inhibition. However, instead of optimizing for high oral bioavailability and long half-life, like most clinical and commercial orally administered MEK inhibitors, we optimized for substantial dermal absorption and rapid clearance from systemic circulation with the goal of limiting systemic exposure and toxicities (*19*). From this drug development approach, NFX-179 emerged as the lead therapeutic candidate (fig. S1A). NFX-179 is a highly specific and potent inhibitor of MEK1/2 with an IC_50_ (median inhibitory concentration) of 135 nM in a MEK1 biochemical cascade assay but only a systemic half-life of approximately 1.5 hours in rat following intravenous administration, thus limiting systemic exposure. This is in stark contrast to current FDA-approved oral MEK inhibitors such as trametinib, which has a half-life of 4 days in humans, cobimetinib, which has a half-life of 2.2 days, and selumetinib, which has a half-life of 8 hours (*20*–*22*). The high potency and selectivity of NFX-179 against MEK1/2, good dermal absorption, and short systemic half-life made it an ideal candidate for topical delivery.

### Application of NFX-179 Topical Gel suppresses MAPK signaling in cNF explants ex vivo

Before launching a clinical trial, we first sought to determine whether application of NFX-179 Topical Gel to the surface of intact human epidermis could penetrate human skin and suppress the MAPK pathway in excised cNFs ex vivo. Phosphorylated ERK (p-ERK) suppression was observed in human cNF explants after application of 0.1 and 0.5% NFX-179 Topical Gel but was not observed in cNF explants treated with the vehicle only (fig. S1, B and C). This demonstrates that NFX-179 applied in a topical gel can penetrate human epidermis and suppress the MAPK pathway in cNF tumors, providing proof of concept and rationale to move the drug product into the clinic.

### Clinical trial enrollment and demographics

**Fig. 1.**
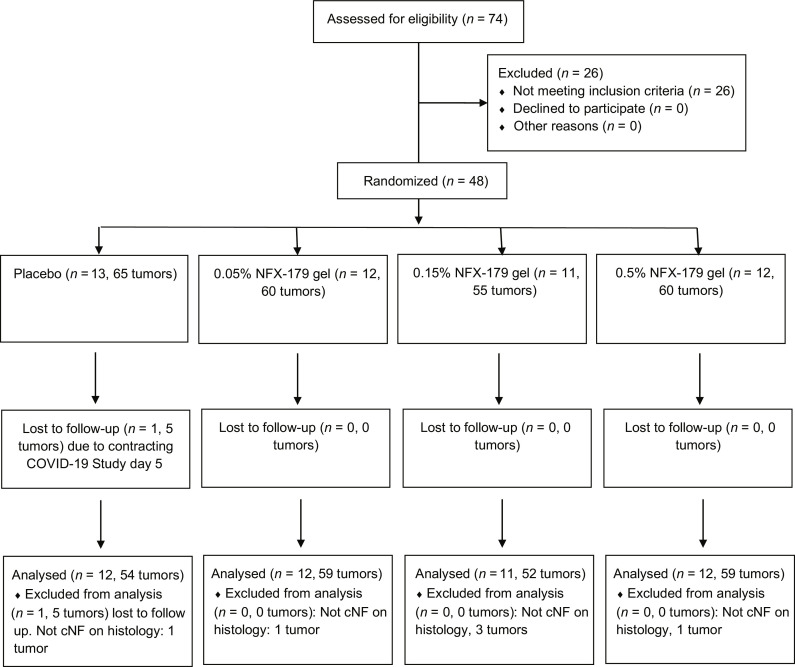
Randomization and follow-up of the participants. Participants were randomized to four treatment arms: vehicle, 0.05% NFX-179 Topical Gel, 0.15% NFX-179 Topical Gel, and 0.5% NFX-179 Topical Gel. Five target tumors were treated with NFX-179 Topical Gel or vehicle once daily during the 28-day treatment period. At day 28 (visit 5), the five Target cNF tumors were measured and then biopsied 4 hours after application for biomarker analysis.

From August 2020 to April 2021, we conducted a multicenter, double-blind, randomized, vehicle-controlled, parallel-group phase 2a study to determine the safety, tolerability, systemic pharmacokinetic, and local pharmacodynamic activity of NFX-179 Topical Gel in persons with NF1 (Fig. 1). Seventy-four persons with a clinical diagnosis of NF1 were screened for eligibility to participate in this clinical trial. Of these, 26 were excluded due to not meeting the eligibility criteria. Ultimately, 48 participants with NF1 (with 240 target cNFs) that met the eligibility criteria were enrolled across six sites in the United States.

Participants were randomly assigned to one of four groups: vehicle (*n* = 13, 65 cNFs), 0.05% NFX-179 Topical Gel (*n* = 12, 60 cNFs), 0.15% NFX-179 Topical Gel (*n* = 11, 55 cNFs), or 0.5% NFX-179 Topical Gel (*n* = 12, 60 cNFs). One participant in the vehicle group did not begin treatment due to contracting COVID-19. The mean age of participants was 47 years (range, 25 to 73 years), with 67% of the participants being female (table S1). Of the participants, 81.3% were white, 10.4% were Black, and 8.3% were Asian.

Eligible tumors for inclusion in the trial were dome-shaped, were nonirritated, and had a baseline diameter between 5 and 10 mm and a height (thickness) of at least 2 mm. At baseline, the mean tumor volume measured by ruler was 119 mm^3^ (range, 39.3 to 572.6 mm^3^), with 19.6% of tumors located on the face, 33.5% on the trunk, and 46.9% on the arms. Tumor and participant demographics were relatively consistent across the treatment arms (table S1). After 28 days of treatment (visit 5), the five target cNF tumors were measured and excised 4 hours after the last drug application to assess p-ERK levels.

### p-ERK is suppressed in a dose-dependent manner in cNFs treated with NFX-179 Topical Gel as compared with vehicle

**Fig. 2.**
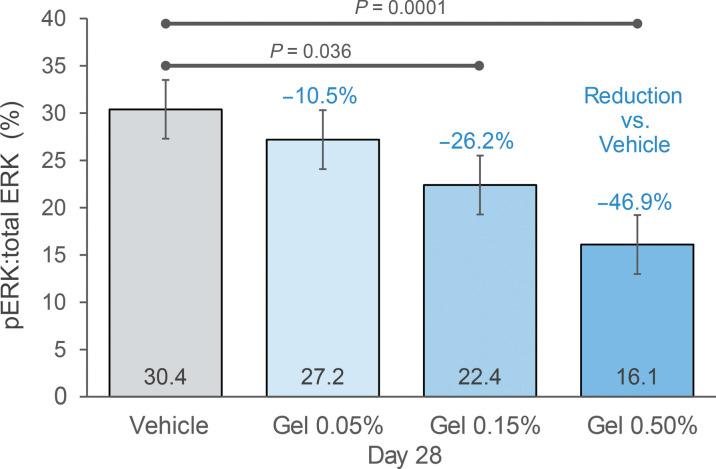
Proportion of p-ERK to total-ERK in cNFs at day 28. Proportion of p-ERK to total ERK in each treatment arm. Numbers in blue are relative to vehicle. 0.15% NFX-179 Topical Gel displayed a mean reduction in p-ERK:total ERK proportion of 26.2% [*P* = 0.036, analysis of variance (ANOVA) analysis]. cNFs treated with 0.5% NFX-179 Topical Gel displayed a mean reduction in p-ERK:total ERK proportion of 46.9% as compared with vehicle (*P* = 0.0001, ANOVA).

A total of 217 cNF biopsies were collected for p-ERK analysis during this study. Eleven target tumors (5%) were excluded from the analysis due to a histopathologic diagnosis inconsistent with cNFs (e.g., intradermal nevus, sebaceous hyperplasia, and cherry angioma). The primary end-point analysis of the remaining 206 cNFs revealed a dose-related reduction in the proportion of p-ERK to total ERK at day 28 with 10.5, 26.2, and 46.9% suppression of p-ERK in the 0.05, 0.15, and 0.5% Gel groups, respectively, as compared to the vehicle group (*P* = 0.38, 0.036, and 0.0001, respectively; Fig. 2 and table S2). In addition, a participant level analysis similarly indicated a 46.7% suppression of p-ERK in individuals treated with the 0.5% NFX-179 Topical Gel as compared to the vehicle group (*P* = 0.0061; table S3).

### Reduction of cNF tumor volume observed after 28 days of treatment with 0.5% NFX-179 Topical Gel as compared with vehicle

**Fig. 3.**
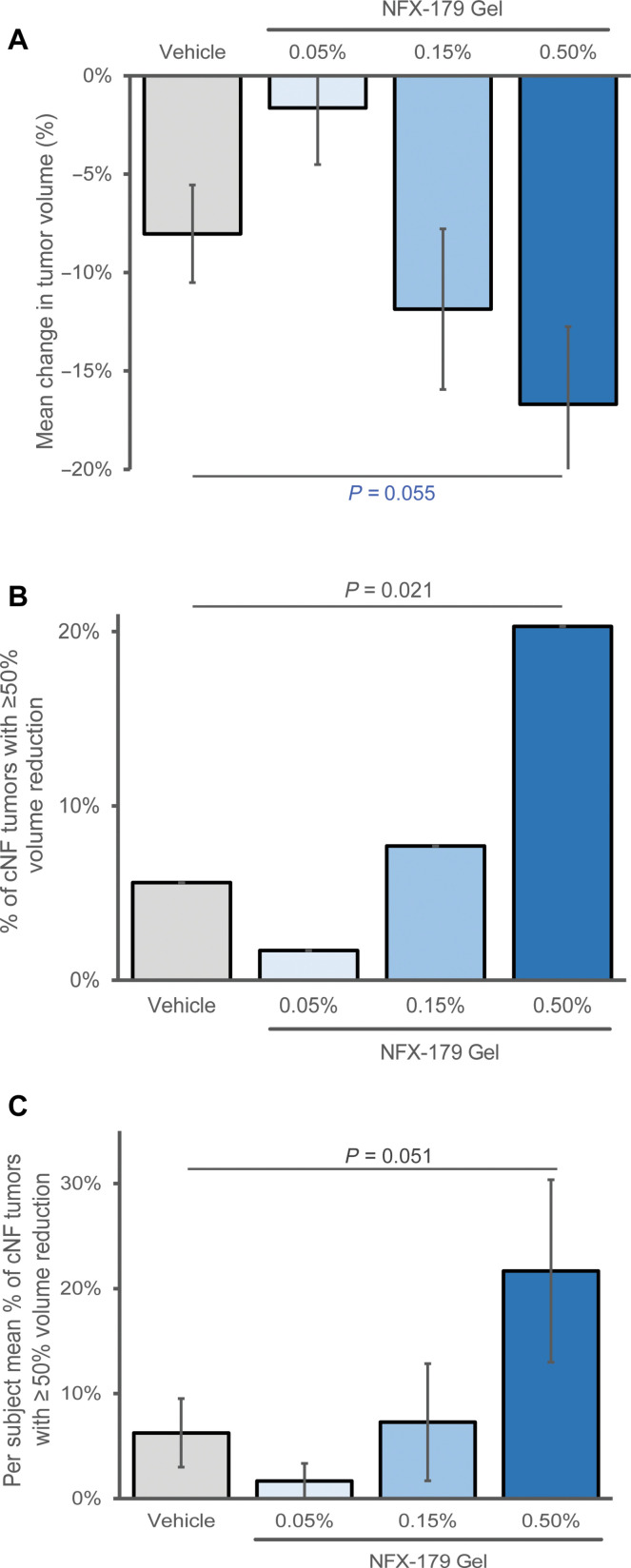
Reduction in volume of cNFs across treatment arms. (**A**) Tumor level analysis: Percent reduction in cNF volume at day 28 compared with baseline in the vehicle group and 0.05, 0.15, and 0.5% Gel groups, respectively. The reduction in cNF volume in 0.5% NFX-179 Topical Gel treatment arm approached significance when compared with vehicle (*P* = 0.055, nonparametric ANOVA analysis). Error bars represent SE. (**B**) Percent of cNF tumors with a 50% or greater reduction in tumor volume. 20% of cNF tumors treated with 0.5% NFX-179 Topical Gel had a ≥50% reduction in volume versus 6% in the vehicle group (*P* = 0.021, ANOVA). (**C**) Participant level analysis: Per participant mean percent of cNFs with 50% or greater reduction in cNF volume. For each participant, the % of the treated cNF tumors with a ≥50% reduction in volume from baseline to day 28 is calculated. There is a 22% per-participant mean response in the 0.5% NFX-179 Topical Gel group versus 6% in vehicle group (*P* = 0.051, ANOVA). The volume of the treated cNFs was calculated on the basis of ruler measurements of longest diameter and height using a calculation of volume as a cylinder.

As we cannot directly measure volume, the volume of the treated cNFs was calculated on the basis of ruler measurements of longest diameter and height using a calculation of volume as a cylinder. Among the 224 cNF tumors analyzed, there was a dose-dependent reduction in tumor volume at day 28, with mean percent change from baseline of −1.6, −11.9, and −16.7% in the 0.05, 0.15, and 0.5% NFX-179 Topical Gel groups, respectively, compared to −8.0% in the vehicle group (Fig. 3A and table S4). The comparison between 0.5% NFX-179 Topical Gel and vehicle approached statistical significance [*P* = 0.055, nonparametric analysis of variance (ANOVA)]. Notably, 20.3% of cNF tumors in the 0.5% NFX-179 Topical Gel arm shrank by 50% or more compared to 5.6% in the vehicle group (*P* = 0.021, nonparametric ANOVA; Fig. 3B and table S5).

In the by-participant responder analysis, the mean percent of tumors with a clinically meaningful 50% or greater reduction from baseline volume was 1.7, 7.3, and 21.7% in the 0.05, 0.15, and 0.5% Gel groups, respectively, compared to 6.3% in the vehicle group (Fig. 3C and table S6). The comparison between 0.5% NFX-179 Topical Gel treatment arm and vehicle approached statistical significance (*P* = 0.051). These findings indicate that suppression of the MAPK pathway by NFX-179 Topical Gel is likely the mechanism underlying reduction in tumor size. Furthermore, our results suggest that p-ERK levels could serve as a useful biomarker for assessing the clinical response to NFX-179 Topical Gel in patients with cNF tumors.

### Reduction of cNF tumor volume correlates with p-ERK suppression

**Fig. 4.**
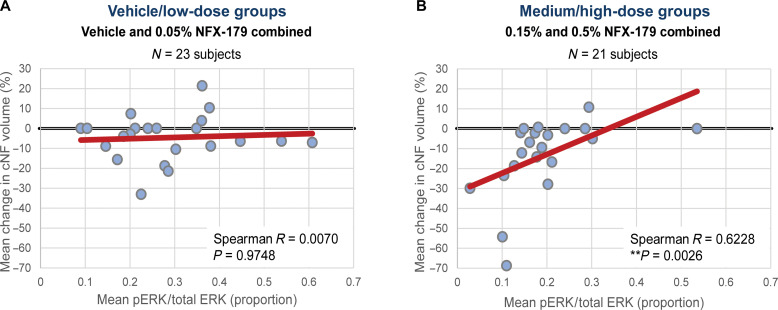
Correlation between percent p-ERK:total-ERK and mean change in cNF volume. Correlation between proportion of p-ERK to total-ERK and mean change in cNF volume. (**A**) In the low-dose 0.05% NFX-179 Topical Gel and vehicle arms, there is no correlation between p-ERK:total ERK proportion and change in tumor volume (Spearman *R* = 0.0070, *P* = 0.9748). (**B**) In the combined medium/high-dose 0.15 and 0.5% NFX-179 Topical Gel treatment arms, there is a moderate correlation between p-ERK:total ERK proportion and change in tumor volume (Spearman *R* = 0.6228, *P* = 0.0026).

To investigate the mechanism of action of NFX-179 Topical Gel in reducing cNF tumor size, we evaluated whether there was a correlation between tumor volume reduction and p-ERK suppression. Our results demonstrate a statistically significant correlation between p-ERK levels and mean percent volume change from baseline to day 28 in the combined 0.15 and 0.5% NFX-179 Topical Gel group (Spearman *R* = 0.63, *P* = 0.001; Fig. 4).

### Minimal to no NFX-179 systemic exposure after 28 days of daily topical application to target cNFs

The pharmacokinetics of NFX-179 was evaluated by measuring plasma concentrations of the drug in a subset of participants (table S7). Plasma samples were collected from four participants, one in the 0.05% Gel group, two in the 0.15% Gel group, and one in the 0.5% Gel group, at visits 2 (pre-dose) and 5 (day 28). On day 28, none of the participants who received 0.05 and 0.15% Gel had detectable levels of NFX-179 in their plasma. The participant who received 0.5% Gel had a plasma concentration of NFX-179 above the limit of quantification of 0.100 ng/ml, at all times, and remained consistent over the 0 to 4 hours post-dose period under 1 ng/ml. These results suggest that after 28 days of daily topical application, there is minimal to no systemic exposure to NFX-179 in participants with cNF tumors.

### NFX-179 is safe and well tolerated

Consistent with the low systemic exposure, no serious adverse effects were reported during the course of the study. Ten adverse events, all of which were mild or moderate, were reported in eight participants—two (15.4%) in the vehicle group, two (16.7%) in the 0.05% NFX-179 Topical Gel group, one (9.1%) in the 0.15% NFX-179 Topical Gel group, and three (25%) in the 0.5% Gel group—but none of these events were attributed to the study drug (table S8). These adverse events included sleep apnea (moderate), lower leg edema (moderate), loss of scalp hair (mild), basal cell carcinoma (moderate, not located in the treatment area), worsening anxiety (moderate), seasonal allergies (mild), COVID-19 infection (two participants: mild and moderate), infection of two excised tumors (mild, procedure related), and symptoms of menopause (mild). Local adverse events were minimal across the treatment arms (tables S9 to S12). No side effects of oral MEK inhibitors (acneiform rash, ocular toxicity, and gastrointestinal toxicity) were observed during the study. There were no adverse events related to changes in clinical laboratory values or electrocardiogram (ECG) findings.

In summary, the results of this first-in-human study indicate that NFX-179 Topical Gel applied once daily for 28 days in participants with cNF tumors is safe and well tolerated at concentrations up to 0.5%. Furthermore, the early clinical and biomarker evidence suggests that NFX 179 Topical Gel may have therapeutic potential in treating NF1 by reducing cNF tumor volume*.*

## DISCUSSION

This clinical trial is the first to evaluate the efficacy and safety of a metabolically labile MEK inhibitor gel, NFX-179, for the topical treatment of cNFs. The primary analysis of the study showed that daily application of NFX-179 Topical Gel on cNFs for 28 days was safe and well tolerated. NFX-179 Topical Gel suppressed p-ERK levels in a dose-dependent manner, with 0.5% NFX-179 Topical Gel reducing p-ERK levels by approximately 47% in target cNFs compared to the vehicle group. This level of p-ERK suppression has been associated with positive clinical response in plexiform neurofibromas–treated oral selumetinib (*23*, *24*), suggesting that it could be a suitable threshold for clinical response in cNFs.

Previous clinical trials have shown that oral selumetinib can cause clinical shrinkage of plexiform neurofibromas, with onset of activity observed no earlier than 4 months, and a median time to response of 8 months (*14*, *15*). In addition, there is a currently enrolling trial of oral selumetinib for cNFs (*25*). An interim report for this study demonstrated at least 20% reduction in average cNF volume compared to baseline in all evaluable participants (*n* = 6). However, these participants also experienced systemic toxicities, including hypertension and rash that limited the duration of treatment.

Oral MEK inhibitors have been shown to cause a variety of adverse events, including rash, diarrhea, peripheral edema, fatigue, dermatitis acneiform, ocular toxicity, and cardiac toxicity. These toxic side effects of oral MEK inhibitors make them less desirable for chronic use in the treatment of cNFs. In contrast, NFX-179 was designed to be rapidly metabolized, limiting systemic exposure and toxicity. As a result, whereas maximum plasma concentrations of selumetinib at the efficacious dose of 25 mg/m^2^ was ~800 ng/ml, the highest exposure after topical administration of NFX-179 Topical Gel was below 1 ng/ml at the top dose, several orders of magnitude lower. Consistent with this, no drug-related adverse events were observed during our study with topical application of NFX-179, and no related systemic toxicities typically associated with MEK inhibitors were observed. In contrast to oral MEK inhibitors, no participants required dose reduction or discontinued treatment due to study drug effects, and no significant difference in adverse events were observed across the treatment arms, demonstrating that topical application of NFX-179 Topical Gel has improved tolerability and reduced adverse effects compared to oral MEK inhibitors.

This 28-day biomarker study was designed to determine the ability of NFX-179 to reach the target tissue in dermis and to inhibit the RAS-MAPK pathway in cNF. Nevertheless, a preplanned analysis of cNF volume was conducted to detect for early evidence of tumor shrinkage. The analysis showed that cNF tumor volume reduced by a mean of 17% from baseline in the 0.5% NFX-179 Topical Gel group compared to an 8% reduction in the vehicle group, demonstrating an early clinical effect on tumor size even in just a 1-month time frame. The data further showed that cNF tumor shrinkage increased with higher doses of NFX-179, indicating that the NFX-179 Topical Gel can shrink the volume of cNF tumors within only 4 weeks of treatment. Note that volume was based on a calculated model using the height and greatest dimension of the cNF as measured by ruler. Ruler-based measurements have been used in clinical trials for assessment of cutaneous skin lesions, including cNFs, and are recommended in the revised RECIST v1.1 criteria (*26*–*29*). Prior work has also evaluated the use of calipers, ultrasound, and three-dimensional photography for cNFs (*28*). Potential variability in use of ruler measurements and the calculated model to estimate volume may be a factor contributing to an 8% reduction in volume observed in the vehicle group, although it is also possible that emollient effect of the vehicle may have improved the thickness of surrounding skin leading to a reduced tumor size measurement. Further work is needed to better define the interrater and intrarater reliability of ruler-based measurements for cNFs.

A recent survey study of 548 adults with NF1 demonstrated that 75% of respondents considered a partial decrease of 33 to 66% in the number or size of their cNFs as a meaningful response (*4*). In our study, we demonstrated that 20% of cNF tumors treated with 0.5% NFX-179 Topical Gel had a 50% or greater reduction in volume compared to only 6% in the vehicle group, demonstrating meaningful clinical response at even 1 month in 20% of tumors in the high dose group. This was highly unexpected given the short time frame of treatment. On the basis of the response timeline from plexiform neurofibromas, we posit that longer treatment with NFX-179 Topical Gel may result in a higher percentage of participants achieving meaningful clinical response.

Biomarker analysis revealed a moderate to high correlation between p-ERK suppression and clinical response. Variability in p-ERK measurements may arise from differences in cellular density within cNFs and variable biopsy sizes affecting tumor and normal skin percentages. Despite these potential sources of variability, a notable moderate-to-high correlation between p-ERK levels and tumor response was identified. The correlation between biomarker suppression and clinical response supports the notion that the suppression of the MAPK pathway by NFX-179 Topical Gel is likely the mechanism that leads to a reduction in tumor size and that higher concentrations in the dermis should be targeted for improved clinical response. A similar association has been demonstrated previously in plexiform neurofibromas (*23*), providing a direct mechanistic connection between NFX-179 Topical Gel–induced p-ERK suppression and cNF tumor shrinkage (*23*). Furthermore, our results suggest that p-ERK levels could serve as a useful biomarker for assessing the clinical response to NFX-179 Topical Gel in patients with cNFs.

We developed and used an ex vivo cNF explant assay before initiating the clinical trial to aid in initial drug screening and formulation and to guide dosing to optimize clinical trial success. The use of fresh cNF explants allowed us to study the effect of NFX-179 in suppressing the RAS/MAPK pathway in target tissue in a rapid and scalable manner. By semi-immersing tissue in media with the epidermis exposed to air, we could assess skin penetration of various topical formulations of NFX-179 and determine dosing levels for target suppression of p-ERK. Our positive clinical trials findings validate this process and demonstrate how ex vivo explants such as those used in this study can support drug development and guide future clinical trial design.

In conclusion, this study demonstrates that topical NFX-179 Topical Gel applied once daily for 28 days in participants with cNFs is safe and well tolerated and can penetrate human skin, suppress p-ERK biomarker, and shrink cNFs after only 28 days of treatment. However, note that the results presented are based on treating a small number of cNFs (*n* = 5) per study participant and for only a 28-day period. In the future, long-term safety, tolerability, and pharmacokinetic studies should be assessed again when treating more tumors simultaneously and when treating patients for more than 28 days. A phase 2b study of 6 months duration in approximately 150 participants is now underway (NCT05005845).

## MATERIALS AND METHODS

### Ex vivo cNF explant analysis

Ex vivo assays were used to determine the activity of NFX-179 Topical Gel in penetrating skin and suppressing the MAPK pathway and to guide dosage choices for the clinical study. NFX-179 Topical Gel 0.1%, 0.5%, or vehicle was applied to the epidermal surface of surgically excised cNF tumors semi-immersed in media with the dermis in media and the surface of the skin exposed for 4 hours (*n* = 3 for each dose). After drug treatment, the tissue was processed for Western blot and immunohistochemistry analysis of p-ERK, a downstream target of the MAPK pathway. Antibodies used included monoclonal rabbit anti–phospho-p44/42 MAPK (Erk1/2) (Thr^202^/Tyr^204^) antibody (Cell Signaling Technology, catalog no. 4370 L), mouse anti–β actin (Sigma-Aldrich, catalog no. A1978), goat anti-mouse immunoglobulin G (IgG) (Jackson ImmunoResearch), and goat anti-rabbit IgG (Thermo Fisher Scientific).

### Participants

From 17 August 2020 to 22 April 2021, we conducted a multicenter, double-blind, randomized, vehicle-controlled, parallel-group phase 2a study to determine the safety, tolerability, pharmacokinetic, and pharmacodynamic activity of 0.05, 0.15, and 0.5% NFX-179 Topical Gel compared with vehicle in participants with cNFs. This study was conducted at six centers in the United States: University of Texas Southwestern Medical Center (Dallas, TX), Center for Dermatology Clinical Research Inc. (Fremont, CA), Minnesota Clinical Study Center (New Brighton, MN), DermResearch Inc. (Austin, TX), Skin Search of Rochester Inc. (Rochester, NY), and The Education & Research Foundation Inc. (Lynchburg, VA).

Persons aged 18 years or older with a clinical diagnosis of NF1 and with at least six cNFs that met study criteria were eligible for the study. Eligible cNFs had the following characteristics: were dome-shaped and discrete; were located on the face, anterior, trunk or upper extremities with a distance at least 5 mm from the orbital rim; had a diameter between 5 to 10 mm and a minimum height of 2 mm; and could not be irritated, infected, or located in an area subject to repeat trauma. Five target cNFs (one on the face and four on the anterior trunk and extremities) were treated with NFX-179 or vehicle; one target cNF (intrasubject control) was not treated. Female participants of childbearing potential were eligible, provided that they had a negative urine pregnancy test and used a protocol-approved, contraceptive method for the duration of the study.

Persons were ineligible for the study if they had applied topical glucocorticoids, alpha-hydroxy acid, fluorouracil, or imiquimod to the target tumor areas in the previous 30 days; had used a MEK inhibitor or BRAF inhibitor in the past 180 days or used a systemic retinoid in the past 90 days. They were also ineligible if they had a history of liver disease, metastatic disease, active cancer, or any other intercurrent illness or physical condition that would impair evaluation of a target cNF.

### Study approval

This trial was conducted under the approval of an FDA Investigational New Drug (IND) application (IND 142734) and the Advarra Institutional Review Board (IRB) (IRB00223615). Written informed consent was obtained from all patients before participation in study procedures. The trial was conducted in accordance with the principles of the Declaration of Helsinki, Good Clinical Practice guidelines, and government regulations. This study is registered with Clinicaltrials.gov (NCT04435665).

### Study design

At the screening visit (visit 1), a full medical history, complete physical exam, ECGs, and safety laboratories were obtained to assess for eligibility. Five target cNF tumors, with one tumor on the face, ranging from 5 to 10 mm in diameter and a minimum height of 2 mm were identified for treatment with study medication. Baseline measurements of target cNFs were obtained using a ruler, and the cNFs were photographed.

At day 1 (visit 2), participants were randomized with a treatment allocation ratio of 1:1:1:1 to the following study medications, vehicle, 0.05% (w/w) NFX-179, 0.15% (w/w) NFX-179, and 0.5% NFX-179, to be applied to the target cNFs once daily during the 28-day treatment period. No other topical treatments other than the study medication were allowed on the target cNFs for the duration of the study. At select clinical sites, blood was also drawn for plasma pharmacokinetic analysis before drug application.

Follow up visits took place at days 8 (visit 3) and 15 (visit 4) and included local tolerability assessments and adverse event assessment. At day 28 (visit 5), the five target cNFs and one untreated cNF were measured then excised 4 (±1) hours after drug application for biomarker analysis. At select clinical sites, blood was also drawn for plasma pharmacokinetics, and samples were collected at 24 (±6) hours after the previous study medication application and just before the last study medication application and at 30 min (±2 min), 1 hour (±4 min), 2 hours (±4 min), and 4 hours (±4 min) after the application completion time.

All participants were also seen between days 36 and 50 (visit 6) for follow-up biopsy care and at day 57 (visit 7) for an end of study visit. Visit 1, 2, and 5 assessments included an ECG and clinical laboratory tests including a complete blood count with differential, alkaline phosphatase alanine aminotransferase, aspartate aminotransferase, blood urea nitrogen, bicarbonate, calcium, chloride, creatinine, glucose, lactate dehydrogenase, phosphorus, potassium, sodium, total bilirubin, total protein, and uric acid.

### Study objectives

The primary objectives of the study were (i) to determine the pharmacodynamic activity of NFX-179 Topical Gel as defined by suppression of p-ERK levels in target cNFs for each NFX-179 Topical Gel group compared with the vehicle gel group after 28 days of daily treatment and (ii) to determine the safety and tolerability of treatment with NFX-179 Topical Gel 0.05, 0.15, and 0.50% when applied once daily for 28 days. The secondary objectives included: to determine the effect of NFX-179 Topical Gel defined as the percent change in cNF tumor volume after 28 days of daily treatment based on tumor volume derived from ruler measurements.

### Study measurements

Levels of the biomarker p-ERK in the excised cNFs were defined as the p-ERK proportion of total ERK in the tumor protein lysate using a size-based nano-immunoassay (the Peggy Sue platform, Protein Simple). Antibodies used included monoclonal rabbit anti–phospho-p44/42 MAPK (Erk1/2) (Thr^202^/Tyr^204^) antibody (Cell Signaling Technology, catalog no. 4370L) and monoclonal rabbit anti-p44/42 MAPK (Erk1/2) antibody (Cell Signaling Technology, catalog no. 4695S). Target tumor diameter and height measured by ruler were the basis of assessments of tumor volume. Volume was calculated on the basis of ruler measurements of longest diameter and height using a calculation of volume as a cylinder with known diameter and height. For systemic pharmacokinetic analysis, samples were collected at one site for evaluation of plasma levels of NFX-179.

### Safety

Treatment emergent adverse events were assessed and coded using MedDRA version 21.0 and a specified local tolerability assessment scale, in which the participant assessed severity of stinging, burning, and pruritus and the investigator assessed the severity of erythema, edema, scabbing/crusting, vesiculation, and erosion. Clinical laboratory evaluations, vital signs, and ECGs were also assessed for abnormalities.

### Statistics

Analyses of efficacy were primarily conducted at the tumor level, under the assumption that tumor responses within the same participant were statistically independent (confirmed by the Shrout-Fleiss intraclass correlation < 0.25). Target cNFs confirmed to be inconsistent with neurofibromas based on histological evaluation of the excised tissue were excluded from the analyses. Pairwise contrasts between each active treatment group versus vehicle were conducted using an ANOVA model. In addition, dichotomized analyses were conducted on the proportion of tumors achieving a clinically meaningful reduction from baseline volume of 50% or greater using Chi-square analysis to compare each active treatment group with the vehicle group.

We also conducted a participant level analysis. For the participant level analyses, the mean p-ERK levels within each participant were first calculated across all five treated tumors. The within-participant mean levels were analyzed using the ANOVA model. In addition, dichotomized analyses were conducted on the mean percent of tumors with a clinically meaningful ≥50% reduction from baseline volume using Chi-square analysis to compare each active treatment group with vehicle. All analyses were done using SAS version 9.4.

## References

[1] R. A. Kallionpaa, E. Uusitalo, J. Leppavirta, M. Poyhonen, S. Peltonen, J. Peltonen, Prevalence of neurofibromatosis type 1 in the Finnish population. Genet. Med. 20, 1082–1086 (2018). 29215653 10.1038/gim.2017.215

[2] G. R. Skuse, B. A. Kosciolek, P. T. Rowley, Molecular genetic analysis of tumors in von Recklinghausen neurofibromatosis: Loss of heterozygosity for chromosome 17. Genes Chromosomes Cancer 1, 36–41 (1989). 2577271 10.1002/gcc.2870010107

[3] P. Wolkenstein, J. Zeller, J. Revuz, E. Ecosse, A. Leplege, Visibility of neurofibromatosis 1 and psychiatric morbidity. Arch. Dermatol. 139, 103–104 (2003). 12533184 10.1001/archderm.139.1.103

[4] A. Cannon, D. C. Pichard, P. L. Wolters, S. Adsit, G. Erickson, A. J. Lessing, P. Li, W. Narmore, C. Rohl, T. Rosser, B. C. Widemann, J. O. Blakeley, S. R. Plotkin, REiNS International Collaboration, Perspective of adults with neurofibromatosis 1 and cutaneous neurofibromas: Implications for clinical trials. Neurology 97 (7 Suppl. 1), S15–S24 (2021). 34230202 10.1212/WNL.0000000000012425 PMC8594006

[5] S. Maguiness, Y. Berman, N. Rubin, M. Dodds, S. R. Plotkin, C. Wong, C. Moertel, REiNS International Collaboration, Measuring the effect of cutaneous neurofibromas on quality of life in neurofibromatosis type 1. Neurology 97, S25–S31 (2021). 10.1212/WNL.0000000000012427 PMC8594000 34230204

[6] D. H. Kim, D. J. Hyun, R. Piquette, C. Beaumont, L. Germain, D. Larouche, 27.12 MHz Radiofrequency ablation for benign cutaneous lesions. Biomed. Res. Int. 2016, 6016943 (2016). 27127789 10.1155/2016/6016943 PMC4835659

[7] S. M. Levine, E. Levine, P. J. Taub, H. Weinberg, Electrosurgical excision technique for the treatment of multiple cutaneous lesions in neurofibromatosis type I. J. Plast. Reconstr. Aesthet. Surg. 61, 958–962 (2008). 17531563 10.1016/j.bjps.2007.03.035

[8] C. Meni, E. Sbidian, J. C. Moreno, S. Lafaye, V. Buffard, S. Goldzal, P. Wolkenstein, L. Valeyrie-Allanore, Treatment of neurofibromas with a carbon dioxide laser: A retrospective cross-sectional study of 106 patients. Dermatology 230, 263–268 (2015). 25662097 10.1159/000368078

[9] L. Q. Le, L. F. Parada, Tumor microenvironment and neurofibromatosis type I: Connecting the GAPs. Oncogene 26, 4609–4616 (2007). 17297459 10.1038/sj.onc.1210261 PMC2760340

[10] N. Ratner, S. J. Miller, A RASopathy gene commonly mutated in cancer: The neurofibromatosis type 1 tumour suppressor. Nat. Rev. Cancer 15, 290–301 (2015). 25877329 10.1038/nrc3911 PMC4822336

[11] J. Mo, S. L. Moye, R. M. McKay, L. Q. Le, Neurofibromin and suppression of tumorigenesis: Beyond the GAP. Oncogene 41, 1235–1251 (2022). 35066574 10.1038/s41388-021-02156-y PMC9063229

[12] E. Jousma, T. A. Rizvi, J. Wu, D. Janhofer, E. Dombi, R. S. Dunn, M. O. Kim, A. R. Masters, D. R. Jones, T. P. Cripe, N. Ratner, Preclinical assessments of the MEK inhibitor PD-0325901 in a mouse model of Neurofibromatosis type 1. Pediatr. Blood Cancer 62, 1709–1716 (2015). 25907661 10.1002/pbc.25546 PMC4546559

[13] W. J. Jessen, S. J. Miller, E. Jousma, J. Wu, T. A. Rizvi, M. E. Brundage, D. Eaves, B. Widemann, M. O. Kim, E. Dombi, J. Sabo, A. Hardiman Dudley, M. Niwa-Kawakita, G. P. Page, M. Giovannini, B. J. Aronow, T. P. Cripe, N. Ratner, MEK inhibition exhibits efficacy in human and mouse neurofibromatosis tumors. J. Clin. Invest. 123, 340–347 (2013). 23221341 10.1172/JCI60578 PMC3533264

[14] E. Dombi, A. Baldwin, L. J. Marcus, M. J. Fisher, B. Weiss, A. Kim, P. Whitcomb, S. Martin, L. E. Aschbacher-Smith, T. A. Rizvi, J. Wu, R. Ershler, P. Wolters, J. Therrien, J. Glod, J. B. Belasco, E. Schorry, A. Brofferio, A. J. Starosta, A. Gillespie, A. L. Doyle, N. Ratner, B. C. Widemann, Activity of selumetinib in neurofibromatosis type 1-related plexiform neurofibromas. N. Engl. J. Med. 375, 2550–2560 (2016). 28029918 10.1056/NEJMoa1605943 PMC5508592

[15] A. M. Gross, P. L. Wolters, E. Dombi, A. Baldwin, P. Whitcomb, M. J. Fisher, B. Weiss, A. Kim, M. Bornhorst, A. C. Shah, S. Martin, M. C. Roderick, D. C. Pichard, A. Carbonell, S. M. Paul, J. Therrien, O. Kapustina, K. Heisey, D. W. Clapp, C. Zhang, C. J. Peer, W. D. Figg, M. Smith, J. Glod, J. O. Blakeley, S. M. Steinberg, D. J. Venzon, L. A. Doyle, B. C. Widemann, Selumetinib in children with inoperable plexiform neurofibromas. N. Engl. J. Med. 382, 1430–1442 (2020). 32187457 10.1056/NEJMoa1912735 PMC7305659

[16] E. C. Pratt, E. Isaac, E. P. Stater, G. Yang, O. Ouerfelli, N. Pillarsetty, J. Grimm, Synthesis of the PET tracer ^124^I-Trametinib for MAPK/ERK kinase distribution and resistance monitoring. J. Nucl. Med. 61, 1845–1850 (2020). 32444378 10.2967/jnumed.120.241901 PMC9364901

[17] C. L. Denton, D. L. Gustafson, Pharmacokinetics and pharmacodynamics of AZD6244 (ARRY-142886) in tumor-bearing nude mice. Cancer Chemother. Pharmacol. 67, 349–360 (2011). 20407895 10.1007/s00280-010-1323-z PMC4332869

[18] K. Y. Sarin, J. Kincaid, B. Sell, J. Shahryari, M. A. J. Duncton, E. Morefield, W. Sun, K. Prieto, O. Chavez-Chiang, C. de Moran Segura, J. Nguyen, R. T. Bronson, S. R. Plotkin, G. G. Kochendoerfer, P. Fenn, M. A. Wootton, C. Powala, M. P. de Souza, K. Y. Tsai, Development of a MEK inhibitor, NFX-179, as a chemoprevention agent for squamous cell carcinoma. Sci. Transl. Med. 15, eade1844 (2023). 37820007 10.1126/scitranslmed.ade1844

[19] Y. Cheng, H. Tian, Current Development Status of MEK Inhibitors. Molecules 22, 1551 (2017). 28954413 10.3390/molecules22101551 PMC6151813

[20] O. Campagne, K. K. Yeo, J. Fangusaro, C. F. Stewart, Clinical pharmacokinetics and pharmacodynamics of selumetinib. Clin. Pharmacokinet. 60, 283–303 (2021). 33354735 10.1007/s40262-020-00967-y PMC7935771

[21] K. Han, J. Y. Jin, M. Marchand, S. Eppler, N. Choong, S. P. Hack, N. Tikoo, R. Bruno, M. Dresser, L. Musib, N. R. Budha, Population pharmacokinetics and dosing implications for cobimetinib in patients with solid tumors. Cancer Chemother. Pharmacol. 76, 917–924 (2015). 26365290 10.1007/s00280-015-2862-0

[22] MEKINIST (trametinib) [package insert] (Novartis, 2022).

[23] G. H. O’Sullivan Coyne, A. M. Gross, E. Dombi, C. Tibery, A. Carbonell, N. Takebe, J. Derdak, D. Pichard, A. K. Srivastava, W. Herrick, R. E. Parchment, S. Martin, P. Wolters, P. Whitcomb, L. Rubinstein, J. H. Doroshow, A. P. Chen, B. C. Widemann, Phase II trial of the MEK 1/2 inhibitor selumetinib (AZD6244, ARRY-142886 Hydrogen Sulfate) in adults with neurofibromatosis type 1 (NF1) and inoperable plexiform neurofibromas (PN), American Society of Clinical Oncology, Virtual Meeting, 29 May to 2 June 2020.

[24] C. f. M. P. f. H. U. (CHMP), "Koselugo (selumetinib)" (2021).

[25] A. Cannon, L. Haynes, T. Skelton, D. Pichard, B. Widemann, B. Korf, Shrinking cutaneos neurofibromas in NF1 with selumetinib, paper presented at the Neurofibromatosis Conference, San Francisco, CA, 21 to 24 September 2019.

[26] E. A. Eisenhauer, P. Therasse, J. Bogaerts, L. H. Schwartz, D. Sargent, R. Ford, J. Dancey, S. Arbuck, S. Gwyther, M. Mooney, L. Rubinstein, L. Shankar, L. Dodd, R. Kaplan, D. Lacombe, J. Verweij, New response evaluation criteria in solid tumours: Revised RECIST guideline (version 1.1). Eur. J. Cancer 45, 228–247 (2009). 19097774 10.1016/j.ejca.2008.10.026

[27] N. Basset-Seguin, A. Hauschild, J. J. Grob, R. Kunstfeld, B. Dreno, L. Mortier, P. A. Ascierto, L. Licitra, C. Dutriaux, L. Thomas, T. Jouary, N. Meyer, B. Guillot, R. Dummer, K. Fife, D. S. Ernst, S. Williams, A. Fittipaldo, I. Xynos, J. Hansson, Vismodegib in patients with advanced basal cell carcinoma (STEVIE): A pre-planned interim analysis of an international, open-label trial. Lancet Oncol. 16, 729–736 (2015). 25981813 10.1016/S1470-2045(15)70198-1

[28] R. D. Thalheimer, V. L. Merker, K. I. Ly, A. Champlain, J. Sawaya, N. L. Askenazi, H. P. Herr, J. L. W. Da, J. T. Jordan, A. Muzikansky, E. M. Pearce, F. H. Sakamoto, J. O. Blakeley, R. R. Anderson, S. R. Plotkin, REiNS International Collaboration, Validating techniques for measurement of cutaneous neurofibromas: Recommendations for clinical trials. Neurology 97, S32–S41 (2021). 34230197 10.1212/WNL.0000000000012428

[29] M. Wataya-Kaneda, Y. Watanabe, A. Nakamura, K. Yamamoto, K. Okada, S. Maeda, K. Nimura, K. Saga, I. Katayama, Pilot study for the treatment of cutaneous neurofibromas in neurofibromatosis type 1 patients using topical sirolimus gel. J. Am. Acad. Dermatol. 88, 877–880 (2023). 36334988 10.1016/j.jaad.2022.08.066

