# Supplementary material for: Effect of NFX-179 MEK inhibitor on cutaneous neurofibromas in persons with neurofibromatosis type 1
Source: Sci Adv. 2024 May 1;10(18):eadk4946. doi: 10.1126/sciadv.adk4946 (PMC11062565; doi:10.1126/sciadv.adk4946)
Supplement: Supplementary file 1 — Fig. S1 Tables S1 to S12 Supplementary Methods [file sciadv.adk4946_sm.pdf]

Science Advances
AAAS

Supplementary Materials for

Effect of NFX-179 MEK inhibitor on cutaneous neurofibromas in persons with neurofibromatosis type 1

Kavita Y. Sarin *et al.*

Corresponding author: Kavita Y. Sarin, ksarin@stanford.edu

Sci. Adv. **10**, eadk4946 (2024)
DOI: 10.1126/sciadv.adk4946

This PDF file includes:

Fig. S1
Tables S1 to S12
Supplementary Methods

Supplementary Material

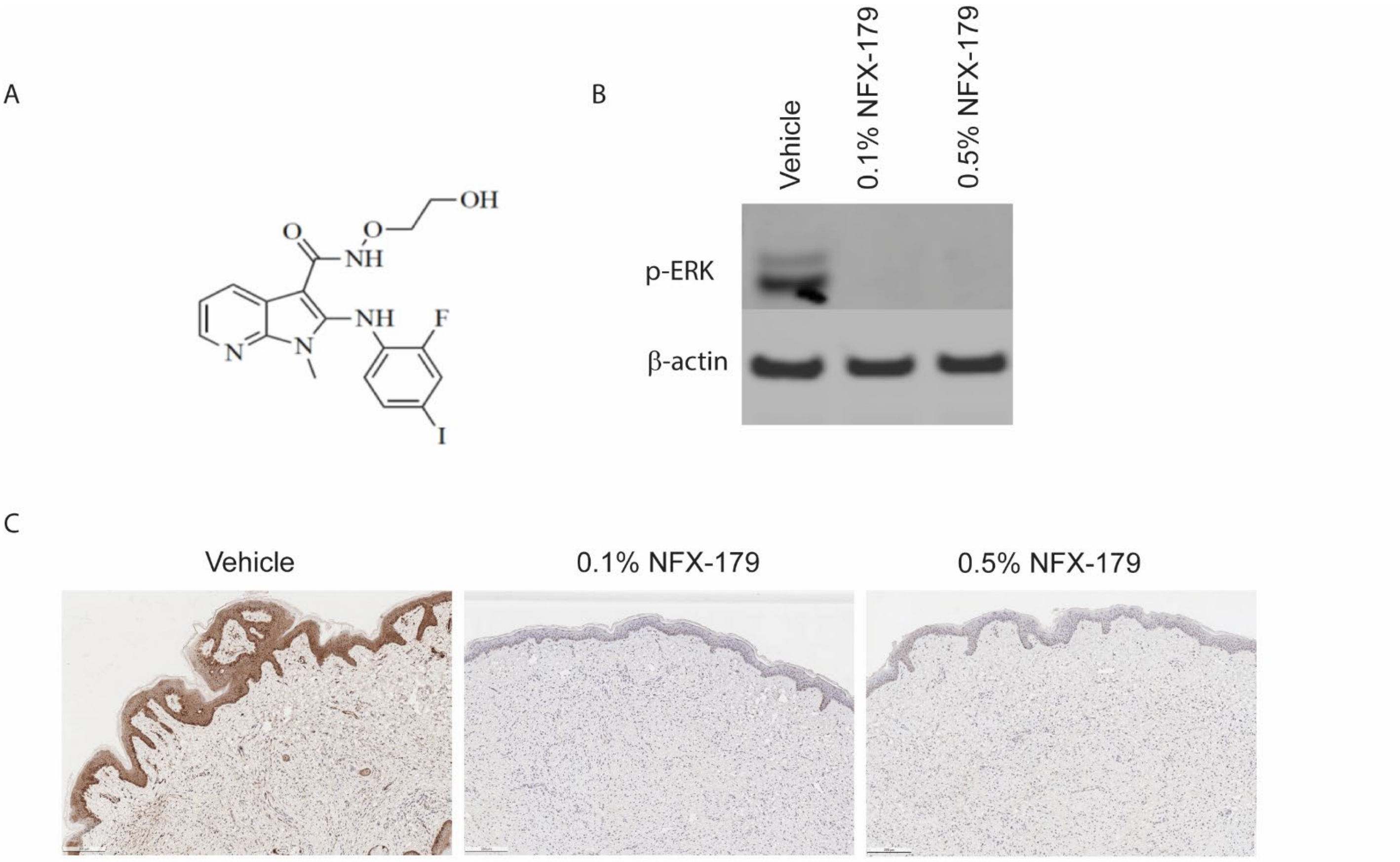

Supplementary Figure 1. NFX-179 Suppresses p-ERK levels in cutaneous neurofibromas in an ex-vivo explant assay. (A) Chemical structure of NFX-179 (B) pERK levels assessed by Western blot in cNF explants treated with two doses of NFX-179 Topical Gel or vehicle. C) Immunohistochemistry analysis of p-ERK levels (brown) in 0.1% and 0.5% NFX-179 treated cNF explants as compared with vehicle (20X).

Supplementary Table 1. Study Demographics

Characteristic	Vehicle N = 13	NFX-179 Gel			Total N = 48
		0.05% N = 12	0.15% N = 11	0.5% N = 12	
Age, years					
Mean (SD)	45.2 (9.84)	51.1 (11.90)	49.5 (16.47)	43.5 (9.92)	47.2 (12.18)
Range	33 – 72	31 – 69	29 – 73	25 – 63	25 – 73
Age Group, n (%)					
19 – 64	12 (92.3)	10 (83.3)	8 (72.7)	12 (100)	42 (87.5)
≥ 65	1 (7.7)	2 (16.7)	3 (27.3)	0	6 (12.5)
Gender, n (%)					
Male	6 (46.2)	3 (25.0)	3 (27.3)	4 (33.3)	16 (33.3)
Female	7 (53.8)	9 (75.0)	8 (72.7)	8 (66.7)	32 (66.7)
Race Category, n (%)					
Non-white	2 (15.4)	1 (8.3)	4 (36.4)	2 (16.7)	9 (18.8)
White	11 (84.6)	11 (91.7)	7 (63.6)	10 (83.3)	39 (81.3)
Race, n (%)					
Asian	1 (7.7)	0	3 (27.3)	0	4 (8.3)
Black/African American	1 (7.7)	1 (8.3)	1 (9.1)	2 (16.7)	5 (10.4)
White	11 (84.6)	11 (91.7)	7 (63.6)	10 (83.3)	39 (81.3)
Ethnicity, n (%)					
Hispanic or Latino	0	2 (16.7)	0	1 (8.3)	3 (6.3)
Not Hispanic or Latino	11 (84.6)	9 (75.0)	7 (63.6)	9 (75.0)	36 (75.0)
Not reported	2 (15.4)	1 (8.3)	4 (36.4)	2 (16.7)	9 (18.8)

Supplementary Table 2. p-ERK proportion of total ERK (%) at Day 28: Tumor level analysis

Statistic	Vehicle	NFX-179 Gel		
		0.05%	0.15%	0.5%
N	53	53	47	53
Mean (SD)	30.36 (21.15)	27.19 (23.82)	22.42 (16.17)	16.14 (11.39)
Median	23.11	21.13	18.28	14.85
Range	5.03 – 98.23	6.22 – 170.39	4.43 – 87.39	0.69 – 49.13
p-value		0.3864	0.0363	0.0001

Supplementary Table 3. p-ERK Proportion of Total-ERK (%) at Day 28: Subject Level Analysis

Statistic	Vehicle	NFX-179 Gel 0.05%	NFX-179 Gel 0.15%	NFX-179 Gel 0.5%
N	12	11	10	11
Mean (SD)	30.54 (13.69)	27.34 (12.57)	22.67 (12.72)	16.27 (6.86)
Median	28.1	27.78	20.22	17.34
Range	10.43-60.76	9.01-53.86	10.07-53.52	2.83-28.52
p-value		0.5189	0.1268	0.0061

Supplementary Table 4. Change in cNF Volume (%) at Day 28: Tumor Level Analysis

Statistic	Vehicle	NFX-179 Gel 0.05%	NFX-179 Gel 0.15%	NFX-179 Gel 0.5%
N	54	59	52	59
Mean Baseline Tumor Volume (SD)	106.8 (75)	139 (107)	108 (95)	120 (96)
Mean Percent Change in Volume (SD)	-8.0 (18.2)	-1.6 (22.1)	-11.9 (29.4)	-16.7 (30.3)
Range	-65.3 - 30	-50 - 85	-93.7 - 80	-88 - 104
p-value		0.377	0.512	0.055

Supplementary Table 5. Percent tumors with 50% or Greater Volume Reduction at Day 28

Statistic	Vehicle	NFX-179 Gel 0.05%	NFX-179 Gel 0.15%	NFX-179 Gel 0.5%
N	54	59	52	59
Tumors with 50% or greater reduction in Volume at Day 28 compared to Baseline (%)	3 (5.6%)	1 (1.7%)	4 (7.7%)	12 (20.3%)
Chi-square p-value		0.27	0.66	0.02

Supplementary Table 6. Mean percent tumors with 50% or Greater Volume Reduction at Day 28: Subject Level Analysis

Statistic	Vehicle	NFX-179 Gel 0.05%	NFX-179 Gel 0.15%	NFX-179 Gel 0.5%
N	12	12	11	12
Mean Percent of Tumors with 50% or greater reduction in Volume at Day 28 compared to Baseline	6.3 (11.3)	1.67 (5.8)	7.3 (18.5)	21.7 (30.1)
Chi-square p-value		0.55	0.9	0.05

Supplementary Table 7. Mean Plasma Concentrations (ng/mL) after NFX-179 Gel Treatment

Visit	Time (h)	Treatment 0.05% NFX-179 (N = 1)	0.15% NFX-179 (N = 2)	0.5% NFX-179 (N = 1)
2	0	BQL	BQL	BQL
5	0	BQL	BQL	0.439
	30 min	BQL	BQL	0.887
	1	BQL	BQL	0.573
	2	BQL	BQL	0.718
	4	BQL	BQL	0.528

BQL - Below the Quantifiable Limit (0.100 ng/mL).

Supplementary Table 8. Number (%) of Subjects with Treatment-Emergent Adverse Events

Adverse Event Category	Vehicle N = 13	NFX-179 Gel 0.05% N = 12	0.15% N = 11	0.5% N = 12
General disorders and administration site conditions				
Oedema peripheral	0	0	1 (9.1)	0
Immune system disorders				
Multiple allergies	0	0	0	1 (8.3)
Infections and infestations				
Postoperative wound infection	0	0	0	1 (8.3)
Investigations				
Corona test positive	2 (15.4)	0	0	0
Psychiatric disorders				
Anxiety	0	1 (8.3)	0	0
Respiratory, thoracic and mediastinal disorders				
Sleep apnoea syndrome	0	0	1 (9.1)	0
Skin and subcutaneous tissue disorders				
Alopecia	0	0	0	1 (8.3)
Basal cell carcinoma	0	1 (8.3)	0	0
Social circumstances				
Menopause	0	0	0	1 (8.3)

MedDRA Version 21.0.

Supplementary Table 9. Local Tolerability Assessment for Erosion

Treatment Group	Sign/Symptom	Visit	N	Grade 0 (None)	Grade 1 (Mild)	Grade 2 (Moderate)	Grade 3 (Severe)
NFX-179 Gel 0.50%	Erosion	Visit 2 (Pre-Med)	60	60 (100.0%)	0 (0.0%)	0 (0.0%)	0 (0.0%)
	Erosion	Visit 2 (Post-Med)	60	60 (100.0%)	0 (0.0%)	0 (0.0%)	0 (0.0%)
	Erosion	Visit 3	60	60 (100.0%)	0 (0.0%)	0 (0.0%)	0 (0.0%)
	Erosion	Visit 4	60	60 (100.0%)	0 (0.0%)	0 (0.0%)	0 (0.0%)
	Erosion	Visit 5 (Pre-Med)	60	60 (100.0%)	0 (0.0%)	0 (0.0%)	0 (0.0%)
	Erosion	Visit 5 (Post-Med)	60	60 (100.0%)	0 (0.0%)	0 (0.0%)	0 (0.0%)
NFX-179 Gel 0.15%	Erosion	Visit 2 (Pre-Med)	55	55 (100.0%)	0 (0.0%)	0 (0.0%)	0 (0.0%)
	Erosion	Visit 2 (Post-Med)	55	55 (100.0%)	0 (0.0%)	0 (0.0%)	0 (0.0%)
	Erosion	Visit 3	55	55 (100.0%)	0 (0.0%)	0 (0.0%)	0 (0.0%)
	Erosion	Visit 4	50	50 (100.0%)	0 (0.0%)	0 (0.0%)	0 (0.0%)
	Erosion	Visit 5 (Pre-Med)	55	55 (100.0%)	0 (0.0%)	0 (0.0%)	0 (0.0%)
	Erosion	Visit 5 (Post-Med)	55	55 (100.0%)	0 (0.0%)	0 (0.0%)	0 (0.0%)
NFX-179 Gel 0.05%	Erosion	Visit 2 (Pre-Med)	60	60 (100.0%)	0 (0.0%)	0 (0.0%)	0 (0.0%)
	Erosion	Visit 2 (Post-Med)	60	60 (100.0%)	0 (0.0%)	0 (0.0%)	0 (0.0%)
	Erosion	Visit 3	60	60 (100.0%)	0 (0.0%)	0 (0.0%)	0 (0.0%)
	Erosion	Visit 4	60	60 (100.0%)	0 (0.0%)	0 (0.0%)	0 (0.0%)
	Erosion	Visit 5 (Pre-Med)	60	60 (100.0%)	0 (0.0%)	0 (0.0%)	0 (0.0%)
	Erosion	Visit 5 (Post-Med)	60	60 (100.0%)	0 (0.0%)	0 (0.0%)	0 (0.0%)
NFX-179 Gel Vehicle	Erosion	Visit 2 (Pre-Med)	65	65 (100.0%)	0 (0.0%)	0 (0.0%)	0 (0.0%)
	Erosion	Visit 2 (Post-Med)	65	65 (100.0%)	0 (0.0%)	0 (0.0%)	0 (0.0%)
	Erosion	Visit 3	60	60 (100.0%)	0 (0.0%)	0 (0.0%)	0 (0.0%)
	Erosion	Visit 4	60	60 (100.0%)	0 (0.0%)	0 (0.0%)	0 (0.0%)
	Erosion	Visit 5 (Pre-Med)	60	60 (100.0%)	0 (0.0%)	0 (0.0%)	0 (0.0%)
	Erosion	Visit 5 (Post-Med)	60	60 (100.0%)	0 (0.0%)	0 (0.0%)	0 (0.0%)

Supplementary Table 10. Local Tolerability Assessment for Erythema

Treatment Group	Sign/Symptom	Visit	N	Grade 0 (None)	Grade 1 (Mild)	Grade 2 (Moderate)	Grade 3 (Severe)
NFX-179 Gel 0.50%	Erythema	Visit 2 (Pre-Med)	60	60 (100.0%)	0 (0.0%)	0 (0.0%)	0 (0.0%)
	Erythema	Visit 2 (Post-Med)	60	60 (100.0%)	0 (0.0%)	0 (0.0%)	0 (0.0%)
	Erythema	Visit 3	60	60 (100.0%)	0 (0.0%)	0 (0.0%)	0 (0.0%)
	Erythema	Visit 4	60	60 (100.0%)	0 (0.0%)	0 (0.0%)	0 (0.0%)
	Erythema	Visit 5 (Pre-Med)	60	59 (98.3%)	0 (0.0%)	1 (1.7%)	0 (0.0%)
	Erythema	Visit 5 (Post-Med)	60	59 (98.3%)	1 (1.7%)	0 (0.0%)	0 (0.0%)
NFX-179 Gel 0.15%	Erythema	Visit 2 (Pre-Med)	55	55 (100.0%)	0 (0.0%)	0 (0.0%)	0 (0.0%)
	Erythema	Visit 2 (Post-Med)	55	54 (98.2%)	1 (1.8%)	0 (0.0%)	0 (0.0%)
	Erythema	Visit 3	55	55 (100.0%)	0 (0.0%)	0 (0.0%)	0 (0.0%)
	Erythema	Visit 4	50	50 (100.0%)	0 (0.0%)	0 (0.0%)	0 (0.0%)
	Erythema	Visit 5 (Pre-Med)	55	55 (100.0%)	0 (0.0%)	0 (0.0%)	0 (0.0%)
	Erythema	Visit 5 (Post-Med)	55	55 (100.0%)	0 (0.0%)	0 (0.0%)	0 (0.0%)
NFX-179 Gel 0.05%	Erythema	Visit 2 (Pre-Med)	60	60 (100.0%)	0 (0.0%)	0 (0.0%)	0 (0.0%)
	Erythema	Visit 2 (Post-Med)	60	60 (100.0%)	0 (0.0%)	0 (0.0%)	0 (0.0%)
	Erythema	Visit 3	60	59 (98.3%)	1 (1.7%)	0 (0.0%)	0 (0.0%)
	Erythema	Visit 4	60	59 (98.3%)	1 (1.7%)	0 (0.0%)	0 (0.0%)
	Erythema	Visit 5 (Pre-Med)	60	60 (100.0%)	0 (0.0%)	0 (0.0%)	0 (0.0%)
	Erythema	Visit 5 (Post-Med)	60	60 (100.0%)	0 (0.0%)	0 (0.0%)	0 (0.0%)
NFX-179 Gel Vehicle	Erythema	Visit 2 (Pre-Med)	65	65 (100.0%)	0 (0.0%)	0 (0.0%)	0 (0.0%)
	Erythema	Visit 2 (Post-Med)	65	65 (100.0%)	0 (0.0%)	0 (0.0%)	0 (0.0%)
	Erythema	Visit 3	60	60 (100.0%)	0 (0.0%)	0 (0.0%)	0 (0.0%)
	Erythema	Visit 4	60	60 (100.0%)	0 (0.0%)	0 (0.0%)	0 (0.0%)
	Erythema	Visit 5 (Pre-Med)	60	60 (100.0%)	0 (0.0%)	0 (0.0%)	0 (0.0%)
	Erythema	Visit 5 (Post-Med)	60	60 (100.0%)	0 (0.0%)	0 (0.0%)	0 (0.0%)

Supplementary Table 11. Local Tolerability Assessment for Scabbing and Crusting

Treatment Group	Sign/Symptom	Visit	N	Grade 0 (None)	Grade 1 (Mild)	Grade 2 (Moderate)	Grade 3 (Severe)
NFX-179 Gel 0.50%	Scabbing/Crusting	Visit 2 (Pre-Med)	60	60 (100.0%)	0 (0.0%)	0 (0.0%)	0 (0.0%)
	Scabbing/Crusting	Visit 2 (Post-Med)	60	60 (100.0%)	0 (0.0%)	0 (0.0%)	0 (0.0%)
	Scabbing/Crusting	Visit 3	60	60 (100.0%)	0 (0.0%)	0 (0.0%)	0 (0.0%)
	Scabbing/Crusting	Visit 4	60	60 (100.0%)	0 (0.0%)	0 (0.0%)	0 (0.0%)
	Scabbing/Crusting	Visit 5 (Pre-Med)	60	59 (98.3%)	1 (1.7%)	0 (0.0%)	0 (0.0%)
	Scabbing/Crusting	Visit 5 (Post-Med)	60	60 (100.0%)	0 (0.0%)	0 (0.0%)	0 (0.0%)
NFX-179 Gel 0.15%	Scabbing/Crusting	Visit 2 (Pre-Med)	55	55 (100.0%)	0 (0.0%)	0 (0.0%)	0 (0.0%)
	Scabbing/Crusting	Visit 2 (Post-Med)	55	55 (100.0%)	0 (0.0%)	0 (0.0%)	0 (0.0%)
	Scabbing/Crusting	Visit 3	55	55 (100.0%)	0 (0.0%)	0 (0.0%)	0 (0.0%)
	Scabbing/Crusting	Visit 4	50	50 (100.0%)	0 (0.0%)	0 (0.0%)	0 (0.0%)
	Scabbing/Crusting	Visit 5 (Pre-Med)	55	55 (100.0%)	0 (0.0%)	0 (0.0%)	0 (0.0%)
	Scabbing/Crusting	Visit 5 (Post-Med)	55	55 (100.0%)	0 (0.0%)	0 (0.0%)	0 (0.0%)
NFX-179 Gel 0.05%	Scabbing/Crusting	Visit 2 (Pre-Med)	60	60 (100.0%)	0 (0.0%)	0 (0.0%)	0 (0.0%)
	Scabbing/Crusting	Visit 2 (Post-Med)	60	60 (100.0%)	0 (0.0%)	0 (0.0%)	0 (0.0%)
	Scabbing/Crusting	Visit 3	60	60 (100.0%)	0 (0.0%)	0 (0.0%)	0 (0.0%)
	Scabbing/Crusting	Visit 4	60	60 (100.0%)	0 (0.0%)	0 (0.0%)	0 (0.0%)
	Scabbing/Crusting	Visit 5 (Pre-Med)	60	60 (100.0%)	0 (0.0%)	0 (0.0%)	0 (0.0%)
	Scabbing/Crusting	Visit 5 (Post-Med)	60	60 (100.0%)	0 (0.0%)	0 (0.0%)	0 (0.0%)
NFX-179 Gel Vehicle	Scabbing/Crusting	Visit 2 (Pre-Med)	65	65 (100.0%)	0 (0.0%)	0 (0.0%)	0 (0.0%)
	Scabbing/Crusting	Visit 2 (Post-Med)	65	65 (100.0%)	0 (0.0%)	0 (0.0%)	0 (0.0%)
	Scabbing/Crusting	Visit 3	60	60 (100.0%)	0 (0.0%)	0 (0.0%)	0 (0.0%)
	Scabbing/Crusting	Visit 4	60	60 (100.0%)	0 (0.0%)	0 (0.0%)	0 (0.0%)
	Scabbing/Crusting	Visit 5 (Pre-Med)	60	60 (100.0%)	0 (0.0%)	0 (0.0%)	0 (0.0%)
	Scabbing/Crusting	Visit 5 (Post-Med)	60	60 (100.0%)	0 (0.0%)	0 (0.0%)	0 (0.0%)

Supplementary Table 12. Local Tolerability Assessment for Vesiculation

Treatment Group	Sign/Symptom	Visit	N	Grade 0 (None)	Grade 1 (Mild)	Grade 2 (Moderate)	Grade 3 (Severe)
NFX-179 Gel 0.50%	Vesiculation	Visit 2 (Pre-Med)	60	60 (100.0%)	0 (0.0%)	0 (0.0%)	0 (0.0%)
	Vesiculation	Visit 2 (Post-Med)	60	60 (100.0%)	0 (0.0%)	0 (0.0%)	0 (0.0%)
	Vesiculation	Visit 3	60	60 (100.0%)	0 (0.0%)	0 (0.0%)	0 (0.0%)
	Vesiculation	Visit 4	60	60 (100.0%)	0 (0.0%)	0 (0.0%)	0 (0.0%)
	Vesiculation	Visit 5 (Pre-Med)	60	60 (100.0%)	0 (0.0%)	0 (0.0%)	0 (0.0%)
	Vesiculation	Visit 5 (Post-Med)	60	60 (100.0%)	0 (0.0%)	0 (0.0%)	0 (0.0%)
NFX-179 Gel 0.15%	Vesiculation	Visit 2 (Pre-Med)	55	55 (100.0%)	0 (0.0%)	0 (0.0%)	0 (0.0%)
	Vesiculation	Visit 2 (Post-Med)	55	55 (100.0%)	0 (0.0%)	0 (0.0%)	0 (0.0%)
	Vesiculation	Visit 3	55	55 (100.0%)	0 (0.0%)	0 (0.0%)	0 (0.0%)
	Vesiculation	Visit 4	50	50 (100.0%)	0 (0.0%)	0 (0.0%)	0 (0.0%)
	Vesiculation	Visit 5 (Pre-Med)	55	55 (100.0%)	0 (0.0%)	0 (0.0%)	0 (0.0%)
	Vesiculation	Visit 5 (Post-Med)	55	55 (100.0%)	0 (0.0%)	0 (0.0%)	0 (0.0%)
NFX-179 Gel 0.05%	Vesiculation	Visit 2 (Pre-Med)	60	60 (100.0%)	0 (0.0%)	0 (0.0%)	0 (0.0%)
	Vesiculation	Visit 2 (Post-Med)	60	60 (100.0%)	0 (0.0%)	0 (0.0%)	0 (0.0%)
	Vesiculation	Visit 3	60	60 (100.0%)	0 (0.0%)	0 (0.0%)	0 (0.0%)
	Vesiculation	Visit 4	60	60 (100.0%)	0 (0.0%)	0 (0.0%)	0 (0.0%)
	Vesiculation	Visit 5 (Pre-Med)	60	60 (100.0%)	0 (0.0%)	0 (0.0%)	0 (0.0%)
	Vesiculation	Visit 5 (Post-Med)	60	60 (100.0%)	0 (0.0%)	0 (0.0%)	0 (0.0%)
NFX-179 Gel Vehicle	Vesiculation	Visit 2 (Pre-Med)	65	65 (100.0%)	0 (0.0%)	0 (0.0%)	0 (0.0%)
	Vesiculation	Visit 2 (Post-Med)	65	65 (100.0%)	0 (0.0%)	0 (0.0%)	0 (0.0%)
	Vesiculation	Visit 3	60	60 (100.0%)	0 (0.0%)	0 (0.0%)	0 (0.0%)
	Vesiculation	Visit 4	60	60 (100.0%)	0 (0.0%)	0 (0.0%)	0 (0.0%)
	Vesiculation	Visit 5 (Pre-Med)	60	60 (100.0%)	0 (0.0%)	0 (0.0%)	0 (0.0%)
	Vesiculation	Visit 5 (Post-Med)	60	60 (100.0%)	0 (0.0%)	0 (0.0%)	0 (0.0%)

Supplementary Methods

Immunohistochemistry

Immunohistochemistry for p-ERK was performed by HistoWiz Inc. (Brooklyn, NY) using standard operating procedures and fully automated workflow. Samples were processed, embedded in paraffin, and sectioned at 4 μm. Immunohistochemistry was performed on a Bond Rx autostainer (Leica Biosystems) with enzyme treatment (1:1000) using standard protocols. The primary antibody used was rabbit p-ERK (Cell Signaling, 4307S, 1:100). Bond Polymer Refine anti-rabbit HRP Detection (Leica Biosystems) was used according to manufacturer's protocol. Sections were then counterstained with hematoxylin, dehydrated and film coverslipped using a TissueTek-Prisma and Coverslipper (Sakura). Whole slide scanning (40x) was performed on an Aperio AT2 (Leica Biosystems).
